# Prevalence of Vitamin D Deficiency and Its Relationship with Clinical Outcomes in Patients with Fibromyalgia: a Systematic Review of the Literature

**DOI:** 10.1007/s42399-021-01105-w

**Published:** 2022-01-15

**Authors:** Omar M. E. Ali

**Affiliations:** School of Medical Education, Newcastle University, 2 Faversham Court, Newcastle upon Tyne, NE3 2XN UK

**Keywords:** Fibromyalgia, Chronic pain, Vitamin D deficiency, Hypovitaminosis D

## Abstract

Fibromyalgia is a debilitating chronic condition which poses a therapeutic challenge to the clinician. With a large backlog in patient flow subsequent to the COVID-19 pandemic and rising numbers of patients with post-acute sequelae of COVID-19 (PASC) presenting with fibromyalgia-like clinical features, there is an increasingly pressing need to identify broad cost-effective interventions. Low levels of vitamin D have previously been reported in patients with fibromyalgia, though any causative link has been difficult to establish. A systematic literature review on the association between vitamin D deficiency and fibromyalgia was performed examining retrospective evidence both for and against an association between vitamin D deficiency (VDD) and fibromyalgia and evaluating the therapeutic benefit from supplementation. A group of six studies were selected based on relevance, use of controls, quality of research and citations. Four primary studies assessing the prevalence of VDD in fibromyalgia patients versus controls were evaluated with a total 3,496 subjects. Three included females only and one larger study assessed males. Two (*n* = 313) concluded the presence of a statistically significant association, and two (*n* = 161) found none. Two randomised controlled trials assessing the effect of vitamin D supplementation in a total of 80 subjects found conflicting results, with pain reduction in one and none in the other. It is likely there exists an association between VDD deficiency and fibromyalgia in a large subset of patients, although establishing primary causation is difficult. There is a need for larger randomised controlled trial designs with more effective comparison with healthy subjects and control for confounding factors. Given VDD is a major problem in the general population, we recommend supplementation be recommended by healthcare professionals to fibromyalgia patients for the purpose of maintaining bone health given their potentially increased susceptibility to developing deficiency and its sequelae.

## Introduction

Fibromyalgia is a chronic condition characterised by diffuse musculoskeletal pain lasting at least 3 months and the presence of multiple tender points on physical examination. It affects 5.4% of the UK population[1] and 13.7 times as many females as males.[2] Despite its functional nature, fibromyalgia has severe negative socioeconomic impacts on the patient as it reduces mobility and quality of life in a way similar to chronic inflammatory diseases.[3] Several factors are associated with the pathophysiology of fibromyalgia, but aetiology remains unclear. There is no single effective treatment for fibromyalgia.[4] Whilst initially studied for its effects on bone health and calcium homeostasis, the global effects of vitamin D have recently become the focus of much research as vitamin D receptors (VDR) are increasingly found in multiple tissues.[5] The similarity in symptomatology of vitamin D deficiency (VDD) and chronic widespread pain has led researchers to investigate the relationship between hypovitaminosis D and fibromyalgia. Osteomalacia is the classic manifestation of VDD in adults and presents with widespread bone pain,[6] which can be confused with and therefore misdiagnosed as fibromyalgia, potentially explaining the high prevalence of VDD in some fibromyalgia patient populations and the beneficial effects of supplementation. However, one study found 55% of fibromyalgia patients having severe VDD but none having markers of osteomalacia such as raised alkaline phosphatase, reduced calcium or phosphate.[7] Another mechanism is secondary VDD that develops in fibromyalgia patients due to less time spent outdoors because of pain, depression or restricted mobility.[7] Conversely, there are proposed mechanisms which support an intrinsic association between vitamin D and fibromyalgia pain. Fibromyalgia appears to be related to an imbalance in neurotransmitters and inflammatory pathways in the central nervous system (CNS) resulting in central sensitisation of pain signals.[8] The level of activity of multiple inflammatory pathways associated with chronic pain such as interleukin-4 and TGF-β1 is downregulated in VDD.[8] Vitamin D also suppresses TNF-α in astrocytes and microglia, which has been implicated in peripheral and central sensitisation.[8] With a large backlog in patient flow subsequent to the COVID-19 pandemic and possibly rising numbers of patients with post-acute sequelae of COVID-19 (PASC) presenting with fibromyalgia-like clinical features, there is an increasingly pressing need to identify broad cost-effective interventions. No conclusive causal factor has been identified yet. The aim of this review is to review evidence both for and against an association between VDD and fibromyalgia, followed by an inspection of the evidence for any therapeutic benefit to vitamin D supplementation.

## Methods

A literature review on the association between vitamin D deficiency and fibromyalgia was performed. A database search on Scopus, PubMed and Google Scholar was conducted to look for articles in English with the search terms “vitamin D deficiency”, “fibromyalgia” and “vitamin D supplement”. Six articles were ultimately selected based on relevance, use of controls, quality of research and citations.

Defining VDD and fibromyalgia is problematic; there is no consensus on the cut-off values for VDD or insufficiency as defined by serum 25-hydroxyvitamin D levels (25-OHD). Also, the criteria for fibromyalgia diagnosis, as defined by the American College of Rheumatology (ACR), changed in 2010 (see Table 1). As a result, the vitamin D cut-off values and fibromyalgia diagnosis criteria selected for each study will be described individually.

**Table 1:** American College of Rheumatology criteria for fibromyalgia diagnosis and classification^28,17^

1990 criteria	1. History of widespread pain (defined as pain on right and left side of the body, above and below the waist for a period of ≥ 3 consecutive months) 2. ≥ 11 out of 18 tender points described as “painful” upon digital palpation
2010 criteria	“1. Pain and symptoms over the past week, based on the total number of painful areas out of 19 parts of the body PLUS level of severity of these symptoms: a. Fatigue b. Waking unrefreshed c. Cognitive (memory or thought) problems 2. Symptoms lasting at least three months at a similar level 3. No other health problem that would explain the pain and other symptoms”

## VDD is not associated with Fibromyalgia

**Fig. 1:**
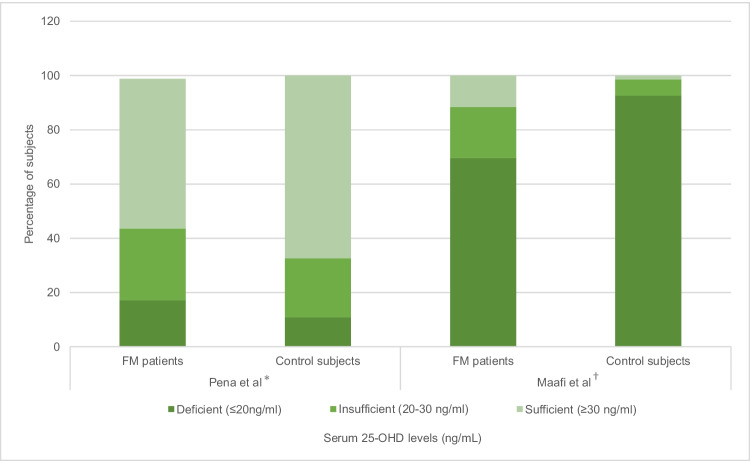
Serum 25-hydroxyvitamin D (25-OHD) levels in fibromyalgia patients and controls. Both studies used the same 25-OHD cut-off values. *(*p* = 0.78). †(*p* = 0.001). Adapted from references(9, 10) FM = fibromyalgia. 25-OHD = 25-hydroxyvitamin D

In a cross-sectional study,[9] Pena et al. evaluated 25-OHD levels in 87 fibromyalgia patients (according to 1990 ACR criteria) compared to 92 age- and sex-matched controls. All participants were female. There was no statistically significant difference in mean serum 25-OHD concentrations between patients and subjects (37.51 ng/mL and 38.23 ng/mL respectively), and no difference after classification of 25-OHD levels as deficient (≤ 20 ng/mL), insufficient (21–30 ng/mL) or sufficient (31–60 ng/mL). In another cross-sectional study by Maafi et al.,[10] 74 female fibromyalgia patients (according to 1990 and 2010 criteria) were compared to 68 control subjects. Unexpectedly, fibromyalgia patients had higher mean serum 25-OHD levels than control subjects (17.24 ng/mL and 9.91 ng/mL respectively) and serum 25-OHD did not correlate with tender point count. See Fig. 1.

Both studies found no association between VDD and fibromyalgia because no statistically significant difference was observed between patients and controls in Pena et al.’s study (*p* = 0.78), and fibromyalgia patients had significantly higher serum 25-OHD in Maafi et al.’s study (*p* = 0.001). This difference was explained by the possibility that fibromyalgia patients took over-the-counter vitamin D supplements due to their pain. No significant correlation between 25-OHD concentrations and pain intensity or the number of positive tender points was observed, reinforcing the conclusion that VDD is not linked to fibromyalgia. Maafi et al. also recorded no association between 25-OHD levels and self-reported fatigue and anxiety scores derived from the Fibromyalgia Impact Questionnaire (FIQ).

Whilst both studies would suggest no benefit in supplementing fibromyalgia patients, VDD was prevalent in patients and controls, especially in Maafi et al.’s study, suggesting a need for population-wide vitamin D supplementation. This is particularly significant given the increase in parathyroid hormone levels observed by Pena et al. with decreasing 25-OHD concentrations, which means there is a greater risk of bone turnover, impaired bone mineralisation and bone density loss.[11] One study concluded that low serum 25-OHD levels was significantly associated with reduced postural stability and an increased risk of falls in fibromyalgia patients.[12] Combined with bone density loss, this places patients at risk of fracture.

The study populations in both studies were similar and relatively large compared to previous cross-sectional studies.[13] Although all subjects were female, the results of these studies remain important since fibromyalgia is significantly more prevalent in women. However, both studies showed selection bias as subjects were selected from secondary care units and rheumatology outpatient clinics. Therefore, results do not reflect the general population of fibromyalgia patients who are restricted to primary care. Furthermore, controls in Maafi et al.’s study were selected from the patients’ families. Estimates of VDD’s heritability from twin studies range from 23 to 80%,[14] so family members of vitamin D deficient fibromyalgia patients may have a greater probability of also having VDD, minimising the observed difference between patients and controls.

Many confounding factors were controlled in Pena et al.’s study, such as BMI, vitamin supplements, mental health and comorbidities affecting vitamin D metabolism, which were not controlled for in Maafi et al.’s study (Table 2). However, neither study controlled for seasonal change. The photochemical synthesis of vitamin D from 7-dehydrocholesterol requires UV-B sunlight. Maximal vitamin D production is observed in summer, with little or no vitamin D produced in winter months depending on latitude.[15] In Pena et al.’s study, data collection occurred during winter and spring for 70.64% of controls and only 54% of patients. Therefore, the actual 25-OHD levels for patients may have been lower than observed had the same proportion of patients been enrolled in winter and spring. In Maafi et al.’s study, the authors did not discuss whether seasons were accounted for. They also did not discuss clothing, which is particularly important in Maafi et al.’s Iranian female population, where cultural dress may reduce exposure to sunlight.[16].

**Table 2:** Factors affecting the comparison of studies

• Accounting for different confounding factors
• Small sample sizes limiting external validity
• Sample variations in ethnicity, sun exposure, latitude of study location
• Cross-sectional design cannot establish whether suboptimal vitamin D status increases fibromyalgia risk, or whether behavioural changes secondary to fibromyalgia result in suboptimal vitamin D levels
• 25-OHD is considered the best measure of vitamin D status in the past 3–4 weeks.^29^ It therefore may not represent the vitamin D status of subjects at the time fibromyalgia first developed

Pena et al. used 1990 ACR criteria for fibromyalgia diagnosis, whereas Maafi et al. used both 1990 and 2010 criteria. The 2010 criteria abolished the need for positive tender points and instead included symptom severity scales and widespread pain, increasing specificity by encompassing patients who had a negative tender point test and therefore did not fit the 1990 criteria for fibromyalgia diagnosis.[17, 18] However, the proportion of patients diagnosed with each set of criteria was not specified, so it is unclear to what extent the greater specificity of the 2010 criteria affected the study population.

## VDD is associated with Fibromyalgia

Olama et al. studied vitamin D levels and bone mineral density (BMD) in 50 premenopausal Egyptian fibromyalgia patients (ACR 1990 criteria) and 50 age-matched healthy female controls between May and July 2010.[19] There was no significant difference in BMI between the two groups. Patients with fibromyalgia had significantly lower serum 25-OHD levels than controls (15.1 and 18.8 ng/ml respectively, *p* = 0.0018) and showed a greater prevalence of deficiency. BMD at the lumbar spine was significantly lower in fibromyalgia patients, and at the femoral neck and radius spine, it showed no significant difference. Fibromyalgia patients were more likely to have impaired short-term memory, depression, sleep and mood disturbance.

**Fig. 2:**
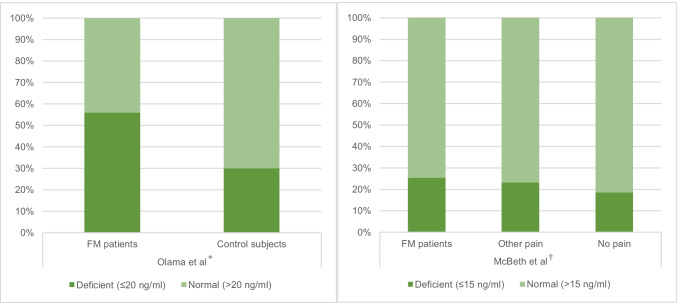
Prevalence of Vitamin D deficiency in patients and controls in Olama et al.’s (left) and McBeth et al.’s (right) study. *(*p* = 0.002). †(*p* < 0.005). Adapted from references(19, 20) FM = fibromyalgia

McBeth et al. carried out a large cross-sectional study encompassing 3075 men from the European Male Ageing Study (EMAS), of which 263 reported fibromyalgia and 1550 reported “other pain” that did not satisfy the 1990 ACR criteria, and 1262 reported no pain.[20] Pain ascertainment and demographic information were assessed by postal questionnaire, and subjects were subsequently invited for serum 25-OHD measurement. In comparison to pain-free patients, patients with fibromyalgia and “other pain” had significantly lower 25-OHD levels (see Fig. 2Figure 2). After age adjustment, fibromyalgia patients had a 50% increase in the odds of having 25-OHD deficiency compared to subjects with no pain, whereas having “other” pain was associated with a 30% increase. These odds ratios persisted after adjustment for physical activity, BMI and depression.

Both studies concluded that VDD is associated with fibromyalgia. This relationship could be explained by musculoskeletal pain or depression in fibromyalgia reducing physical activity and hence reducing exposure to sunlight. Indeed, one cross-sectional study found VDD to be associated with anxiety and depression in fibromyalgia patients,[21] which would warrant a programme to promote physical activity and counselling to fibromyalgia patients to increase vitamin D levels. However, results from the EMAS suggest that this is not the case, as adjusting for physical activity did not eliminate the odds ratio of having VDD, indicating a possible intrinsic relationship between vitamin D and pain.

Olama et al. demonstrated that VDD in fibromyalgia is a risk factor for developing osteoporosis as it reduces BMD in the lumbar spine. A nutritional programme rich in calcium and vitamin D is necessary to prevent bone loss and reduce fractures in fibromyalgia patients.[22] Whilst screening all fibromyalgia patients for BMD loss by dual-energy X-ray absorptiometry would be expensive, promoting calcium and vitamin D supplementation can be a prophylactic measure.

There was little difference in VDD prevalence between patients with fibromyalgia and “other pain” in McBeth et al.’s study (see Fig. 2). Although the exact nature of what classifies “other pain” was not clarified, the relationship between vitamin D and pain in general deserves further research.

The study population size of the EMAS study was one of the largest yet to investigate the relationship between vitamin D and fibromyalgia (*n* = 3075, male). One limitation of the EMAS study was the pain assessment method; pain history and location were ascertained by self-reported postal questionnaires, introducing reporting bias if patients do not reveal accurate information regarding pain severity. This is pertinent in this male study population as males are more likely to withhold pain-related information.[23] Olama et al. ascertained pain by physical examination, reducing the possibility of variation between patients. However, tender point count was measured using digital palpation with enough pressure “to blanch a nail”. This digital palpation method is less reliable and reproducible than other methods such as pressure dolorimetry, as the former can be affected by hand temperature.[24].

Whilst Olama et al. used the standard definition of VDD as ≤ 20 ng/ml,[25] McBeth et al. used a cut-off of ≤ 15 ng/ml, as this was the value below which the frequency of fibromyalgia increased. As a result, it is natural that they would conclude an association between low vitamin D and fibromyalgia. If a cut-off of 20 ng/ml was implemented, the relationship would be slightly attenuated. Nonetheless, this does not affect the validity of the conclusion that hypovitaminosis D was more prevalent in fibromyalgia patients.

## Effect of Vitamin D Supplementation on Fibromyalgia

In a randomised control trial (RCT) by Warner et al.,[26] 50 female fibromyalgia patients with serum 25-OHD between 9 and 20 ng/ml were divided into two equal groups which were either supplemented with 50,000 IU of ergocalciferol weekly (also known as vitamin D2) or placebo for 3 months. Pain was assessed using the visual analogue scale (VAS) and functional pain score (FPS). After 3 months, there was no statistically significant difference in VAS scores between the treatment and control group, and the placebo group reported lower FPS scores than the treatment group.

Wepner et al. [27] randomised 30 female fibromyalgia patients with serum 25-OHD < 32 ng/ml into two equal groups, but, unlike Warner et al., supplementation involved 1200–2400 IU cholecalciferol (vitamin D3) depending on baseline serum 25-OHD levels. Supplementation lasted 20 weeks after which subjects were evaluated, and were re-evaluated after 24 weeks without supplementation. A significant reduction was observed in pain intensity as measured by VAS in the treatment group compared to the control group. Twenty-four weeks after ceasing supplementation, the difference in 25-OHD levels diminished between the treatment and control groups as did the reduction in pain intensity. However, no significant differences were found with respect to anxiety and depression.

In Warner et al.’s study, ergocalciferol was used for 3 months, achieving an increase in average 25-OHD levels from 16.8 to 31.3 ng/ml. In contrast, Wepner et al. used cholecalciferol, and within 3 months observed an increase in average 25-OHD from 19.0 to 50.96 ng/ml, demonstrating a more significant increase with cholecalciferol. This confirms the result of a recent meta-analysis that found cholecalciferol to be more effective in raising serum 25-OHD than ergocalciferol[28] and is therefore the preferred vitamer for supplementation.

Warner et al. concluded that correction of VDD did not alleviate musculoskeletal pain, whereas Wepner et al. found it to be a safe and cheap treatment for pain in fibromyalgia. However, Wepner et al. indicated no benefit of vitamin D supplementation on mental health symptoms or health-related quality of life. The results did indicate a significantly better outcome regarding morning fatigue, and this was reinforced by the fact that the pattern of reduction of morning fatigue corresponded to that of pain intensity as measured by VAS. This selective response indicates that fibromyalgia is an extensive syndrome in which VDD may only be a contributing factor.

Wepner et al. found seemingly contradicting outcomes. Although the treatment group experienced a significant reduction in pain intensity compared to the control group, there was no correlation between doses of Vitamin D3 and the change in pain severity within the treatment group itself, even though different subjects received different doses depending on their baseline vitamin D status. The selectivity of this response invites further research into the effect of vitamin D on fibromyalgia-specific pain rather than general chronic widespread pain.

Both trials randomised patients in a double-blinded manner, reducing the possibility of observer bias (the tendency for researchers to see a reduced pain score in the treatment group) and performance bias (the tendency for researchers to give more attention to the treatment group and therefore more care, ancillary treatment, etc.), which would reduce the validity of their conclusions. However, the method of allocation of patients to either the treatment or control group in Warner et al.’s study was not revealed, which may skew the outcomes if dissimilar groups were generated. However, baseline characteristics of each group demonstrated no significant difference between the treatment and placebo group with respect to age, ethnicity, serum 25-OHD, pain duration and intensity. Wepner et al. adjusted for BMI also, whereas Warner et al. notably did not assess subject body weight. Obesity has been consistently associated with lower concentrations of 25-OHD and obese/overweight individuals may require greater supplementation levels to achieve adequate serum vitamin D levels, potentially contributing to the reduced efficacy of supplementation observed in their study.[29].

Both RCT’s had a small sample size, limiting external validity. Although Wepner et al. used a smaller sample than Warner et al. (*n* = 30 and 50 respectively), the former invited patients from the general population and local fibromyalgia support groups, not just secondary care settings where patients may have multiple comorbidities which confound the data, and therefore is more indicative of the general fibromyalgia population.

A longer treatment period was implemented by Wepner et al. (20 weeks as opposed to 12 weeks in Warner et al.), which allowed for a more accurate indication of the long-term effect of vitamin D supplementation. Wepner et al. also followed up patients after 24 weeks of no supplementation, allowing them to assess whether any effects observed after 24 weeks were treatment-specific. This was not undertaken by Warner et al. The latter restricted treatment to 3 months, based on the resolution of bone pain in biopsy-proven osteomalacia within 1–3 months,[7] not the resolution of muscle pain. A longer trial period could allow muscle fibre regeneration and a reduction in pain.

## Conclusion

There is conflicting evidence regarding the association between vitamin D and fibromyalgia (see Table 3). Only studies which used control groups were involved in this report, though there are many other conflicting studies which lacked control groups,[13] and therefore could not compare VDD prevalence in fibromyalgia patients to healthy populations. A limitation of all the studies assessing the association between VDD and fibromyalgia was their retrospective or cross-sectional nature. A long-term prospective cohort study can more accurately evaluate the association, because it allows baseline exposure status before deficiency occurs to be determined and reduces the risk of other confounding variables.

**Table 3:** Summary of research studies used in this report^10, 11 19, 20, 26, 27^

*Author and year*	Study type	Subjects	Duration	Serum vitamin D assessment	Test criteria	Conclusions
*Pena *et al*. (2010)*	Cross-sectional	87 fibromyalgia patients + 92 controls All female	Nov 2007–Jan 2009	25-OHD High-performance liquid chromatography ≤ 20 = deficient 21–30 = insufficient 31–60 = sufficient	ACR^a^ 1990 criteria	Deficient and insufficient vitamin D not observed more frequently in fibromyalgia patients No association between VDD^b^/insufficiency and pain intensity
*Maafi *et al*. (2016)*	Cross-sectional	74 fibromyalgia patients + 68 controls All female	April 2013–Sep 2013	25-OHD Chemiluminescence immunoassay (CLIA) kit ≤ 20 = deficient 20–30 = insufficient ≥ 30 = normal	ACR 1990 or 2010 criteria	High prevalence of VDD in patients and controls but no statistically significant difference between the two groups
*Olama *et al*. (2012)*	Cross-sectional	50 fibromyalgia patients + 50 controls All female	May 2010–Jul 2010	25-OHD ELISA immunoassay ≤ 8 = severely deficient ≤ 20 = deficient > 20 = not deficient	ACR 1990 criteria Assessed pain intensity, clinical severity, depression, sleep disturbance and BMD	Higher prevalence of deficiency in patients All other variables showed worse outcomes in fibromyalgia patients except BMD^c^ in femoral neck and radius spine (no difference)
*McBeth *et al*. (2010)*	Cross-sectional	263 fibromyalgia patients + 1550 patients with “other pain” + 1262 pain-free subjects All male	2003–2009	25-OHD Equilibrium radioimmunoassay < 15 = deficient > 15 = not deficient	ACR 1990 criteria	Patients with fibromyalgia and “other pain” had increased odds of VDD. Slightly attenuated by body mass index (BMI) and depression
*Warner *et al*. (2008)*	RCT^g^	50 patients with VDD (20 placebo + 22 receiving 50,000 IU^d^ ergocalciferol) All female	Patients recruited between May–Aug 2004 Treatment duration: 3 months	25-OHD Liquid chromatography tandem mass spectrometry ≤ 20 = deficient > 20 = not deficient	ACR 1990 criteria (but did not necessitate the presence of > 11 tender points)	Supplementation had no effect on pain compared to baseline, and no effect on pain at 3 months compared to placebo group
*Wepner *et al*. (2014)*	RCT	30 patients with VDD (15 in placebo group + 15 receiving 1200–2400 IU cholecalciferol) All female	Treatment duration: 20 weeks Follow up: 24 weeks post-treatment	25-OHD Assay not outlined < 32 = deficient	ACR 1990 + 2010 criteria	Supplementation reduced pain intensity. No effect on depression, FIQ^e^ scores and SF-36^f^ physical health component

^a^American College of Rheumatology. ^b^Vitamin D deficiency. ^c^Bone mineral density. ^d^International units. ^e^Fibromyalgia Impact Questionnaire. ^f^36-Item Short Form Health Survey. ^g^randomised controlled trial

Evidence regarding the effect of supplementation on quality of life in fibromyalgia patients was also inconclusive, with both RCT’s reaching different results. Sample sizes were small, necessitating a larger RCT involving different ethnicities and latitudes that can be more representative of the global fibromyalgia population and account for the seasonal, cultural and dietary habits that have a major impact on vitamin D status. A “stratified” designed randomised controlled trial is suggested for future research, whereby fibromyalgia patients are stratified into vitamin D replete and deficient at baseline for the purpose of analysis to determine whether the effect of the intervention differs in the two groups whilst still being conducted in a blinded manner.

In conclusion, evidence linking the prevalence of VDD and the effect of vitamin D supplementation in fibromyalgia patients is inconclusive, with evidence and mechanisms to support a case for and against. Nonetheless, VDD is a major problem in the general population and supplementation should be recommended by GP’s to fibromyalgia patients for the purpose of maintaining bone health given their increased susceptibility to developing deficiency.

## Data Availability

Data sharing is not applicable to this article as no datasets were generated or analysed during the current study.
